# Prospective Evaluation of Extracorporeal Shockwave Lithotripsy in Renal and Upper Ureteric Stone Treatment: Clinical Assessment and Results

**DOI:** 10.7759/cureus.61102

**Published:** 2024-05-26

**Authors:** Nimisha Singh, Shikhar Agarwal, Rajeev Sarpal

**Affiliations:** 1 General Surgery, Himalayan Institute of Medical Sciences, Swami Rama Himalayan University, Dehradun, IND; 2 Plastic and Reconstructive Surgery, Jawaharlal Nehru Medical College (JNMC) Aligarh Muslim University (AMU), Aligarh, IND; 3 Urology, Himalayan Institute of Medical Sciences, Swami Rama Himalayan University, Dehradun, IND

**Keywords:** met, hounse field unit, retrograde intra renal surgery (rirs), double j-stent, treatment of urolithiasis, steinstrasse, mini percutaneous nephrolithotomy (mini-pcnl), percutaneous nephrolithotomy (pcnl), eswl (extracorporeal shockwave lithotripsy)

## Abstract

Introduction: Extracorporeal shockwave lithotripsy (ESWL) is a widely accepted non-invasive treatment for renal and upper ureteric stones smaller than 2 cm due to its safety and efficacy. Despite advancements in minimally invasive techniques, extracorporeal shockwave lithotripsy remains an important modality.

Aims and objective: This prospective observational study aimed to evaluate the outcomes of ESWL in managing renal and upper ureteric stones measuring less than 2 cm in terms of stone clearance.

Material and methods: In a study conducted at a university-affiliated tertiary care hospital, 119 patients with renal and upper ureteric stones underwent extracorporeal shockwave lithotripsy over a 12-month period. Data on patient demographics, stone characteristics, treatment procedures, and complications were collected. Follow-up assessments were performed at two-week intervals for up to two months post-treatment.

Results: The mean age of patients was 39.78 years, with a mean stone size of 1.2 cm. Right kidney stones were more prevalent (61.3% [n=76]). Complications included fever (19.3% [n=23]), gross haematuria (24.3% [n=29]), and steinstrasse (21.8% [n=26]). The success rate of extracorporeal shockwave lithotripsy was 81.5% (n=97), with 18.5% (n=22) of patients requiring surgical intervention due to incomplete fragmentation or residual fragments >4 mm. Stone size and density played significant roles in treatment success.

Conclusion: Despite advancements in minimally invasive techniques, ESWL retains its significance as a noninvasive and effective treatment option for renal and upper ureteric stones smaller than 2 cm. Its success depends on various factors, including the stone site, size, and composition. ESWL offers advantages such as minimal morbidity, shorter hospital stays, and better patient compliance. Complications such as steinstrasse are manageable with conservative measures or ancillary procedures. While ESWL may be losing ground in some cases, its noninvasive nature and favourable outcomes make it a valuable option in the armamentarium for stone management.

## Introduction

After urinary tract infection and prostate disease, kidney stones are the leading disease of the genitourinary tract. They are common in all societies, irrespective of socioeconomic status. If not treated properly, 20% of patients will develop some form of kidney dysfunction [1].

The occurrence of kidney stones is reported to be around 5% to 10%. It is widely more common in men than women, and the recurrence rate is around 50% [2]. Familial causes and environmental factors play an important role in its increasing prevalence. Stones composed of calcium oxalate are the most common type, with notably high recurrence rates in males (70-80%) and around 47-60% in females [3]. Medical expulsion therapy (MET) is a non-surgical treatment modality suitable for smaller stones. However, it is ineffective for kidney stones larger than 5 mm. Hence, this underscores the significance of extracorporeal shockwave lithotripsy (ESWL) in fragmenting and expelling such stones.

Nephrolithiasis remains a prevalent condition, particularly in the Himalayan region, known as the "stone belt," and ESWL is a well-established modality for managing kidney and upper ureter stones smaller than 2 cm. Its efficacy depends on several factors. Despite the availability of minimally invasive techniques, extracorporeal shockwave lithotripsy remains important due to its non-invasive nature, safety, low morbidity, and satisfactory stone clearance rate [4]. It is globally accepted as a standard treatment for kidney and upper ureteric stones. Therefore, this study aimed to evaluate the outcomes of extracorporeal shockwave lithotripsy in managing renal and upper ureteric stones measuring less than 2 cm.

## Materials and methods

This prospective observational study, conducted at the Department of General Surgery, Himalayan Institute of Medical Sciences (HIMS), Swami Ram Nagar, Dehradun, aimed to evaluate the outcome of ESWL in managing renal and upper ureteric stones measuring less than 2 cm in terms of stone clearance. Over a 12-month period, a total of 119 patients diagnosed with kidney stone disease were recruited following investigations, including X-ray of the kidney, ureter, and urinary bladder (KUB), ultrasound of the KUB, and non-contrast computed tomography (NCCT) KUB for diagnosis confirmation. Inclusion criteria encompassed patients aged over 18 years with renal and upper ureteric stones less than 20 mm, while exclusion criteria included pregnancy, impacted stones, stone density >1400 HU, gross hydronephrosis, persistent infection, severe hypertension, morbid obesity, and patients on anticoagulants. The data were analyzed using IBM SPSS Statistics Version 24 software (IBM Corp., Somers, NY, USA).

Treatment options were discussed with eligible patients, and those providing informed consent were included in the study. Patients were treated in a supine position, with the stone's location determined using either fluoroscopy or ultrasound. Prior to treatment, patients were instructed to take Bisacodyl (Dulcolax) 10 mg orally and fast until midnight. All treatments were carried out using intravenous analgesia in the form of fentanyl IV (1 μg/kg/dose) and midazolam IV (0.05-0.1 mg/kg) administered during the ESWL procedure. Vital signs, including heart rate (HR), respiratory rate (RR), blood pressure (BP), and oxygen saturation (pulse oximetry), were monitored continuously during the procedure.

Patients underwent ESWL using the Dornier Compact Sigma ESWL machine. A maximum of 3 ESWL sessions, with 2500-3500 shockwaves per session, at intervals of two weeks apart, were allowed. The ESWL settings applied were as follows: For kidney stones, the protocol involved delivering 2500-3000 shockwaves, with a maximum energy level (max) ranging from 3 to 4. The procedure commenced with an initial 100 shockwaves at a level of 0.1-1, followed by gradual escalation to the maximal level. Specifically, the energy level was set at 3.0 for stones located in the lower calyx, 3.5 for those in the upper and middle calyx, and 4.0 for stones situated in the pelvis. The shockwave frequency was maintained at 60 per minute. In the case of ureteric stones, the treatment regimen entailed administering 3000-3500 shockwaves with a maximum energy level of 4, particularly for stones in the upper ureter. Following the treatment, patients were discharged with oral medications, including Diclofenac 50 mg three times daily, Tamsulosin 0.4 mg once daily, and a urinary alkaliniser sachet twice daily for a duration of two weeks.

Descriptive analysis included parameters such as age, sex, stone site, side, number of ESWL sessions, auxiliary and ancillary procedures, and complications. Follow-up assessments were conducted at two-week intervals for up to two months, with X-rays, screening ultrasounds, and NCCT KUB performed to assess for residual calculi. Success was defined as the complete clearance of stones, while residual fragments larger than 4 mm were considered failures.

## Results

The average age of patients with kidney stones is around 40 years, with a wide age range due to the standard deviation of 12 years. The average stone size is 1.2 cm, with variability indicated by the standard deviation of 0.906 cm, suggesting that most stones are between 0.294 cm and 2.106 cm. Kidney stones are more commonly found in the right kidney (61.2% [n=76]) compared to the left kidney (38.7% [n=43]). There is a significantly higher prevalence of kidney stones in males compared to females, with a ratio of 2.7:1. This suggests that males are almost three times more likely to develop kidney stones than females (Table 1). 

**Table 1:** Depicts number of male and female patients and side of stones n: number of patients

Parameters	n	%
Male	82	68.9
Female	37	31.1
Right-side stone	76	61.3
Left side stone	43	38.7

The success of stone clearance varies by location. The pelvi-ureteric junction shows the highest clearance rate at 88.2% (n = 15), followed by the upper ureter at 81.81% (n=27), indicating a more effective treatment or easier access for removal. In contrast, the lower calyx has the lowest clearance rate at 66.6% (n=4), suggesting greater difficulty in clearing stones from this location. The upper calyx and middle calyx have similar and relatively high clearance rates, ranging from 75.0% (n=24) to 75% (n=27). These variations highlight the impact of stone location on the success of stone removal procedures (Table 2).

**Table 2:** Depicts location of stones and stone clearance rate

S. no	Location of stones	Total no. of cases (n = 119)	Total stone clearance in %
1	Upper calyx	27 (22.7%)	24 (77.8%)
2	Middle calyx	36 (30.2%)	27 (75.0%)
3	Lower calyx	6 (5.04%)	4 (66.6%)
4	Pelviureteric junction	17 (14.3%)	15 (88.2%)
5	Upper ureter	33 (27.7%)	27 (81.81%)

Complications

ESWL complications included fever in 19.3% (n=23) and gross haematuria in 24.3% (n=29), all managed with culture-specific antibiotics and adequate hydration. No one reported symptoms or signs of perforation. Steinstrasse with colic, known to occur after fragmentation of a large stone, was observed in 21.8% (n=26) of the cases. Out of these, five required ureteroscopy and lithotripsy, five were managed with a DJ (double-J) stent, and the remaining 16 were managed conservatively with increased hydration, alpha-blockers, analgesics, and antibiotics.

Outcome

Success was confirmed through documented clearance on X-ray KUB and ultrasonography. It was reported to be 81.5% (n=97). All patients with incomplete fragmentation or residual fragments >4 mm were considered failures and underwent surgical procedures such as ureteroscopic lithotripsy (URSL), flexible ureteroscopic lithotripsy, or percutaneous nephrolithotomy (PCNL). A total of 18.5% (n=22) patients did not have a favourable outcome and were termed failures after incomplete or no fragmentation due to factors such as stone location, size, and density. Stone characteristics vary by location: lower calyx stones are fewer but very dense - 1200-1400 HU (Hounsefield unit), indicating harder stones. Pelvis stone has the lowest density (700 HU), indicating a potentially softer stone. Upper calyx and upper ureter stones have moderate size ranges and density variations. Middle calyx stones have the widest size range (0.8-2.0 cm) and high density variability. The majority of stones ranged from 0.8 cm to 2 cm in size, with HU ranging between 780 and 1400. The lower the stone density in HU, the softer the stone composition, the easier it is to disintegrate, and vice versa (Table 3).

**Table 3:** Details on the number, size range, and density range of kidney stones based on their location

Stone location	No. of stones	Stone size in cm (range)	Stone density in HU (range)
Upper calyx	8	0.8–1.2	780–1250
Middle calyx	5	0.8–2.0	800–1400
Lower calyx	2	1	1200–1400
Pelvis	1	1	700
Upper ureter	6	0.8–1.4	842–1400

## Discussion

The clearance rate of successful ESWL ranged between 48% and 85% in most studies, whereas this study reported a success rate of 81.5%. For example, Padhye et al. reported an overall stone-free rate of 91.7% in the upper ureter [5], while Ghimire et al. reported a clearance rate of 91.1% in 112 patients [6]. Al-Marhoon et al. reported success rates of 74% for kidney stones and 88% for ureteric stones [7]. Gupta et al. found a higher stone clearance rate for stones with a diameter of <1.1 cm and mean stone densities of 750 HU, with a stone clearance rate of 90% [8].

The male-to-female ratio varied across different studies. In this study, the male-to-female ratio was 2.7:1. Joshi mentions a ratio of 1.5:1 [9]. Ghayalini et al. report 1:0.3 [10]. The mean age of the patients was 37.2 (SD = ± 10.9) years, and the mean stone size was 7.98 mm (SD = ± 1.18). Wang et al. compared the efficiency of ESWL on different types of stones determined by NCCT [11]. The composition of stones can be determined with the help of a CT scan. When photons pass through an object, they encounter resistance and impediments to their flow. This is dependent on the density of the material and the attenuation of signal strength, which is recorded as HU. It has a scale ranging from −1000 to +1000. Water has an HU value of 0, air stands at −1,000 HU, and dense bone has 1,000 HU. The HU of a stone sheds information about its type because the radiation absorption of each component is different. Most stones in this study ranged between 700 and 1000 HU and were located in the upper ureter, middle calyx, and proximal ureter. Auxiliary procedures such as double J stent insertion were employed in patients presenting with mild to moderate hydronephrosis. This intervention aimed to preserve ureteral patency to facilitate the passage of small or fragmented stones and to preempt potential complications like steinstrasse. While the majority of patients (78.2%) did not necessitate double J stent placement, 21.8% underwent DJ stenting before undergoing ESWL. The stone-free rate for upper, middle, and lower calyx was 77.8% (n=24), 75.0% (n=27), and 66.6 (n=4), respectively, while for pelviureteric junction and upper ureteric stones, it was 88.2% (n=15) and 81.8% (n=27). This was consistent with the study by Hamal et al., which reported 85.9%, 90.25%, and 50.5% success rates for the upper, middle, and lower calyx, respectively [12].

Post-ESWL complications were observed, including fever in 23 cases (19.3%) and gross hematuria in 29 patients (24.3%). Each complication was managed with culture-specific antibiotics and adequate hydration. None of the patients exhibited symptoms or signs of perforation. Steinstrasse with associated colic, a known occurrence following the fragmentation of large stones, manifested in 26 cases (21.8%). Among these, 5 out of 26 necessitated ureteroscopic lithotripsy, while 5 received a DJ stent. The remaining 16 patients were conservatively managed with increased hydration, alpha-blockers, analgesics, and antibiotics. Salem et al. reported common adverse effects such as flank region pain or discomfort and microscopic haematuria, which were effectively alleviated through proper hydration and pain management on an outpatient basis [13]. In a study by Sayed et al., it was noted that steinstrasse was conservatively managed in 48% of patients, while 23% underwent repeat ESWL and 6% required ureteroscopy [14]. Among the 22 patients who experienced failed extracorporeal shockwave lithotripsy due to incomplete or no fragmentation of the stones, ancillary procedures were pursued.

While extracorporeal shockwave lithotripsy (ESWL) offers numerous benefits, it also has notable limitations. First, its effectiveness diminishes for stones with high density or larger sizes, often requiring supplementary procedures for complete stone clearance [15]. Second, ESWL can result in adverse effects such as pain, hematuria, and the risk of steinstrasse. Furthermore, treatment outcomes may be influenced by factors like stone composition, location, and patient anatomy, which are not always accurately predictable. Patient cooperation and tolerance are crucial for successful ESWL, but discomfort during the procedure may lead to poor compliance [16]. Certain stone types, such as cystine or calcium oxalate monohydrate stones, may not respond optimally to ESWL and may necessitate alternative treatment methods [17].

However, it is important to acknowledge the limitations of this study. First, its single-centre design may limit the generalizability of the findings to other settings with different demographic or regional characteristics. Additionally, the use of specific inclusion and exclusion criteria may have introduced selection bias, affecting the applicability of the results. Although the study considered stone size and location, it did not consistently document other critical factors, such as stone composition, which could significantly impact the success rate of ESWL. Moreover, the study employed a specific ESWL machine, the Dornier Compact Sigma, and outcomes may vary with different devices, making it challenging to extrapolate these findings universally.

## Conclusions

Over the last 30 years, the treatment of renal stones has evolved into minimally invasive surgical procedures. However, lower-pole kidney stones and congenital malformations like horseshoe kidneys have poorer outcomes with ESWL. This study reaffirms the efficacy of ESWL as a non-invasive treatment for renal and upper ureteric stones smaller than 2 cm, achieving an overall success rate of 81.5%. Among newer treatment modalities like PCNL, mini percutaneous nephrolithotomy, micro-mini percutaneous nephrolithotomy, retrograde intra renal surgery (RIRS), or URSL, the ESWL offers a balance of efficacy, safety, non-invasiveness, and an acceptable stone-free rate.
